# Biomonitoring of Serum Inorganic Element Concentrations in Morbidly Obese Patients: Impact of Bariatric Surgery

**DOI:** 10.3390/toxics13030152

**Published:** 2025-02-23

**Authors:** Álvaro Ramos-Luzardo, Pilar Fernández Valerón, Beatriz Vanessa Díaz-González, Manuel Zumbado, Katherine Simbaña-Rivera, Inmaculada Bautista-Castaño, Norberto Ruiz-Suárez, Elisabeth Hernández-García, Judith Cornejo-Torre, Octavio P. Luzardo, Lluis Serra-Majem, Luis Alberto Henríquez-Hernández

**Affiliations:** 1 Department of Biochemistry and Molecular Biology, Physiology, Genetics, and Immunology, Universidad de Las Palmas de Gran Canaria, 35016 Las Palmas de Gran Canaria, Spain; alvaro.ramos@ulpgc.es (Á.R.-L.); pilarfdez.valeron@ulpgc.es (P.F.V.); 2 Toxicology Unit, Clinical Sciences Department, Universidad de Las Palmas de Gran Canaria, 35016 Las Palmas de Gran Canaria, Spain; manuel.zumbado@ulpgc.es (M.Z.); katherine.simbana@ulpgc.es (K.S.-R.); norberto.ruiz@ulpgc.es (N.R.-S.); octavio.perez@ulpgc.es (O.P.L.); 3 Research Institute of Biomedical and Health Sciences (IUIBS), Universidad de Las Palmas de Gran Canaria, 35016 Las Palmas de Gran Canaria, Spain; beatrizvanessa@gmail.com (B.V.D.-G.); elisabethernandez924@gmail.com (E.H.-G.); judith.cornejo.dn@gmail.com (J.C.-T.); lluis.serra@ulpgc.es (L.S.-M.); 4 Centro de Investigación para la Salud en América Latina (CISeAL), Facultad de Medicina, Pontificia Universidad Católica del Ecuador (PUCE), Quito 170525, Ecuador; 5 Centro de Investigación Biomédica en Red Fisiopatología de la Obesidad y la Nutrición (CIBEROBN), Instituto de Salud Carlos III, 28029 Madrid, Spain; inmaculada.bautista@ulpgc.es

**Keywords:** obesity, bariatric surgery, inorganic elements, heavy metals, rare-earth elements, weight loss, obesogens

## Abstract

Adipose tissue, in addition to serving as energy storage, can store lipophilic substances, some of which may pose a health risk if massively mobilized during rapid weight loss. This study aimed to biomonitor inorganic elements in obese patients undergoing bariatric surgery and analyze the role of sociodemographic factors. ICP-MS was employed to quantify 55 elements, including elements identified from the ATSDR’s Substance Priority List and rare-earth elements, in serum samples of 58 obese patients before and after bariatric surgery. A total of 39 out of 55 elements were detected, either before or after surgery, in at least one patient. Serum concentrations of gold, copper, mercury, platinum, and selenium significantly decreased after surgery. Serum concentrations of iron and zinc were significantly lower before surgery. Age, sex, diabetes status, arterial hypertension, and family history of obesity were demographic factors associated with the concentration of elements. Correlation analyses showed significant associations between elements and total lipid concentration or body mass index. Our findings indicate a complex interplay between inorganic elements and body fat and provide relevant information regarding the biomonitoring of these chemical elements in a specific and understudied population.

## 1. Introduction

Inorganic elements comprise the entirety of the 118 elements listed on the periodic table [1]. The biological significance of these elements varies widely; some are essential for human health in trace amounts, others have demonstrated deleterious effects on living organisms, and the effects of many remain undetermined. In any case, elevated levels of any of them, either acute or chronic, lead to physiological alterations and the possible development of diseases [2,3]. The group of heavy metals, defined as elements with high atomic weights and densities at least five times greater than that of water, is perhaps the most widely studied. The existing literature on the subject has shown adverse effects on the nervous system, cognition and behavior, skin, and the digestive system, among many others [4,5,6]. The increase in the production and use of electronic devices poses an environmental challenge. It is estimated that more than 50 million tons of electronic waste (e-waste) are produced each year, with recycling rates below 15% [7]. This means that many of the inorganic elements used in the manufacture of electronic devices are dumped into the environment, contaminating soils, water, and food. Among the materials used in the construction of electronic devices, rare-earth elements (REEs) hold particular importance. REEs is a collective term used to designate the 17 chemical elements in the periodic table (the 15 lanthanides, plus scandium and yttrium) characterized by their incompletely filled 4f atomic orbitals, which result in unique magnetic, optical, and chemical properties that are essential for many modern technologies [8]. Unlike other elements, the adverse effects of REEs on human health remain unclear, although it is known that they interact with a wide variety of biomolecules [9] and have been associated with the development of some diseases [10].

Recent research has transformed adipose tissue from a mere biological storage compartment into a metabolically active endocrine organ [11]. Although it plays a protective toxicological role by sequestering lipophilic xenobiotics, adipose tissue also acts as an internal source of chronic exposure due to lipolysis-induced release. Adipocytes express receptors that can be modulated by xenobiotics, reinforcing the obesogen theory [12,13]. Obesogens are substances that contribute to an increased risk of developing overweight and obesity conditions. Persistent organic pollutants are among the most prominent [14], although there is evidence suggesting that some inorganic elements may also be involved [13].

Obesity has doubled globally in the last three decades and has a severe socioeconomic impact [15]. According to the World Health Organization, 60% of European adults are overweight or obese [16]. Obesity is defined by a high body mass index (BMI) and classified as follows: overweight (25–30 kg/m^2^), class I (30–35 kg/m^2^), class II (35–40 kg/m^2^), or class III (≥40 kg/m^2^) obesity [17]. It is caused by a combination of genetic, physiological, behavioral, lifestyle, dietary, and environmental factors. While once primarily linked to fat accumulation, obesity is now understood to involve systemic inflammation, gut microbiota dysregulation, adipocyte cell cycle changes, and oxidative stress [18]. Altogether, these factors destabilize energy homeostasis and increase the risk of obesity [18]. Beyond physical limitations, obesity significantly increases the risk of developing serious health conditions [19,20,21]. Significant advancements in obesity treatment have occurred [22]. Lifestyle interventions, pharmacological therapies (e.g., lipase inhibitors), oral hydrogel capsules, or endoscopic bariatric therapies are the most frequently applied to reduce adiposity [23]. However, while pharmacotherapy shows promising results, bariatric surgery remains the most effective to achieve long-term weight loss and reduce comorbidities [22,24,25]. This type of surgery is typically recommended for poor treatment responses, class II obesity with complications, or class III obesity.

In a recent study, we investigated the behavior of certain organic molecules in a population of morbidly obese individuals undergoing bariatric surgery [26]. The present study aimed to biomonitor serum concentrations of fifty inorganic elements, encompassing essential elements, heavy metals, and rare-earth elements (REEs) in patients subjected to bariatric surgery for the treatment of morbid obesity. This study sought to determine the variations in serum concentrations, elucidate their interactions with adipose tissue, and evaluate their potential contribution to obesogenesis.

## 2. Materials and Methods

### 2.1. Study Population and Sample Collection

A total of 58 adult patients (42 women; 16 men) aged 20–69, with BMIs ranging from 35 to 60 kg/m^2^, were recruited. All participants underwent bariatric surgery following a clinical evaluation between 30 March and 7 July 2015. After surgery, they adhered to a strict dietary regimen for one year and participated in a second evaluation from 15 March to 23 December 2016. Weight, height, and body composition were measured at baseline and follow-up using the Tanita BC-420MA III device (Girod Medical, Madrid, Spain). Additionally, whole-blood samples were collected in 4 mL EDTA-containing tubes and centrifuged at 3000× *g* for 5 min to obtain serum, which was stored at −20 °C for subsequent chemical analysis. Weight loss outcomes were reported as total body weight loss (TBWL), percentage of total body weight loss (%TBWL), and percentage of excess body weight loss (%EWL). Changes in clinically relevant parameters, including lipidemia, smoking habits, comorbidities, and depressive state, were assessed longitudinally.

Ethical approval was obtained from the CHUIMI Ethics Committee (Complejo Hospitalario Universitario Insular Materno Infantil) on 26 June 2014, with the ethical approval code 2024-093-728.

### 2.2. Selection of Elements, Standards, and Sample Preparation

The analysis included 55 elements, comprising elements identified from the ATSDR’s Substance Priority List of 2022 [27], which were selected due to their prevalence, toxicity, and potential for exposure in populations. Rare-earth elements (REEs) and other minor elements (MEs) were included. REEs and MEs are becoming increasingly prevalent in populations globally due to increasing use in technological, medical, and zootechnical sectors [8,28,29]. For the analysis of these elements, we utilized the compilation provided by Tansel (2017) [8] regarding elements used in computers and high-tech devices. The complete list of elements analyzed is detailed in Table S1.

The methodology for the study was adapted from protocols previously established by our research group, as detailed by Henríquez-Hernández in 2023 [30]. Briefly, 0.5 mL of serum samples were weighed using a high-precision analytical balance in a digestion vessel. To these, 2 mL of 65% high-purity nitric acid, distilled in a sub-boiling system equipped with a PTFE-TFM purification unit (SubCLEAN, Milestone SRL, Sorisole, Italy), and 7.5 mL of ultrapure water from a Milli-Q system were added to achieve complete mineralization of the samples. Microwave-assisted extraction (MAE) was conducted using a Milestone Ethos Up apparatus (Milestone, Bologna, Italy). The conditions applied were power (W)–temperature (°C)–time (min): step 1: 1800–100–5; step 2: 1800–150–5; step 3: 1800–200–8; and step 4: 1800–200–7. To verify that the MAE was effective, certified reference materials (Fish Muscle ERM^®^-BB422 (Institute for Reference Materials and Measurements; Geel, Belgium); Lobster Hepatopancreas NRC LUTS-1 (National Research Council; Ottawa, ON, Canada); Seronorm Trace Elements Whole Blood L-1 (SERO AS; Billingstad, Norway)) were added before the procedure. Following extraction, the samples were cooled and transferred into metal-free polypropylene tubes. Subsequently, aliquots of the samples were diluted in autosampler vials to a final concentration of 4% (*v*/*v*). After MAE, the internal standard (ISTD) was manually spiked to facilitate elemental analysis, as previously published [31].

The ISTD solution, containing scandium (Sc), germanium (Ge), rhodium (Rh), and iridium (Ir), each at 20 mg/mL, was introduced into the ICP-MS at a final concentration of 40 ppb. This procedure was carried out to evaluate reproducibility in terms of counts per second (CPS) and to determine the relative standard deviation (RSD) and recovery rates of four isotopes (45Sc, 72Ge, 103Rh, and 193Ir), which should fall within the 70–130% range. Germanium was eliminated from analysis to avoid interferences caused by doubly charged ions of neodymium (Nd) and samarium (Sm). The standards for the purity of elements were acquired as certified reference materials from CPA Chem (Stara Zagora, Bulgaria) and consisted of a multi-element solution of 100 mg/L in 2% nitric acid (HNO_3_), a single-element solution of mercury at 100 mg/L in 5% HNO_3_, and individual solutions for the REEs and MEs at 1000 mg/L in diluted HNO_3_ or HNO_3_/HF for ICP-MS. Consequently, three different calibration curves were prepared using a 4% HNO_3_ solution, except for the mercury calibration curve, which additionally required 1% HCl: (1) a multi-element curve including the main heavy metals, potentially toxic, and some minor elements (nine points ranging from 0.00 to 2000 ppb); (2) a mercury-specific curve (five points ranging from 0.00 to 50 ppb); and (3) a curve for REEs and the majority of MEs (six points from 0.00 to 25 ppb). These levels were chosen based on linearity, sensitivity, and precision to achieve superior analytical parameters and quantification quality. Furthermore, isotopes were selected to minimize interferences from doubly charged ions of REEs. To prevent memory effects, particularly with mercury, a cleaning solution of 2% HNO_3_ and 1% HCl was employed between vials. Given the sample weight of 0.5 g and the subsequent dilution during digestion and analysis, the final matrix concentration in the analyzed sample accounts for 1.5%, suggesting a minimal matrix effect [31].

### 2.3. Analytical Procedure

The analysis was performed using an Agilent 7900 ICP-MS system (Agilent Technologies, Tokyo, Japan), equipped with standard nickel cones, a MicroMist glass concentric nebulizer, and an Ultra High Matrix Introduction (UHMI) system. The Integrated Sample Introduction System (ISIS) was configured for discrete sampling. To address isobaric interferences, specific isotopes were selected to ensure accurate measurements. The 4th generation Octopole Reaction System (ORS4) operated in helium (He) mode for all elements to minimize interferences from low-mass elements, thereby enhancing detection limits. Prior to analysis, a tuning solution containing a mixture of Cs, Co, Li, Mg, Th, and Y was used to optimize instrument performance concerning CPS, RSD values, and the recommended oxide ratio and double ion charges. Each sample was analyzed in triplicate using separate vials, with three complete readings taken automatically for each vial, providing nine individual measurements per sample. Quantification of elements was conducted using MassHunter v.4.2 ICP-MS Data Analysis software by Agilent Technologies.

The entire procedure underwent in-house validation using a certified reference material (Seronorm™ Trace Elements Whole Blood, Billingstad, Norway) at three concentration levels (L-1, L-2, and L-3). The reference material contained clinically relevant concentrations for all elements studied, except In and Ti which were assessed through fortified matrix assays. Five replicates from five independent vials at each level were digested following the sample protocol and analyzed under identical ICP-MS conditions. Linear calibration curves were appropriate for most elements, with regression coefficients (R^2^) ≥ 0.995, and recoveries ranged from 79% to 121% for REEs and MEs, and from 76% to 124% for toxic elements listed by ATSDR. Ten reagent blanks, treated similarly to the samples, were also integrated into the work sequence during the analysis. Instrumental limits of detection (LODs) and quantification (LOQs) were, respectively, determined based on signals three and ten times higher than the average blank solution signal measured on the same day as the sample analysis (Table S1). This approach ensures accurate estimation of LODs and LOQs by accounting for daily instrumental condition variations.

Method accuracy and precision were evaluated through recovery studies using a 4% (*v*/*v*) nitric acid solution spiked with two mixtures at three concentration levels: one containing a multi-elemental mix, and one for the REEs and MEs. Recovery values were calculated by dividing the obtained concentration by the expected theoretical concentration based on the added levels of both solutions. LOQs for determining the sample concentration were calculated by multiplying the instrumental LOQ by the dilution factor applied during the procedure and adjusting for exact mass. All analytical values used for quantification were derived from measurements with RSD < 5%.

Quality control of blood sample analyses included independent vials every 24 sample replicates, each containing a digested blank, two customized mixes, and each level (L-1, L-2, and L-3) of the certified reference material. This practice ensured that analyte concentrations fell within 20% of the RSD, thus verifying the absence of contamination and ensuring accurate and reproducible results.

### 2.4. Statistical Analysis

Descriptive statistics were calculated. Continuous variables were summarized by mean, standard deviation, median, range, and interquartile range (25th and 75th percentiles). Categorical variables were summarized by frequency and proportion. Values below the limit of quantification (LOQ) were considered missing. The normality of continuous variables was assessed using the Shapiro–Wilk test. For variables with detection frequencies exceeding 30%, group comparisons were performed using either paired-sample parametric tests (Student’s *t*-test) or non-parametric alternatives (Mann–Whitney U test and Wilcoxon’s W test), following a Levene’s test to assess the homogeneity of variances and ensure appropriate test selection. This approach was made considering both methodological robustness and public health relevance, and it does not lead to left censoring, as it does not arbitrarily remove low-concentration values and instead prioritizes elements with meaningful prevalence. McNemar’s tests were employed for categorical variable comparisons. Spearman’s or Pearson’s correlation tests were used to assess correlations between inorganic elements and continuous variables.

Given the sample size and the number of variables analyzed, we conducted power calculations for key comparisons, particularly those involving elemental concentrations between exposure groups. The power analysis was based on effect sizes observed in previous studies assessing elemental bioaccumulation, assuming an alpha level of 0.05 and a two-tailed hypothesis. The achieved power (1 − β) for detecting medium-to-large effect sizes (Cohen’s d or Rank Biserial Correlation (rrb) > 0.5) was above 80%, indicating that our study design was sufficiently robust to detect meaningful differences in quantitative variables. For qualitative variables, we applied Cramér’s V to measure the strength of association between categorical variables, ensuring that relationships were statistically meaningful. This approach allowed us to assess the effect size of categorical comparisons while accounting for sample distribution across different groups.

Statistical analyses were performed using Jamovi v2.6.25. Graphs were created using BioRender software (version 4) (Toronto, ON, Canada).

## 3. Results

### 3.1. Influence of Bariatric Surgery on Sociodemographic Variables

After the follow-up (534 ± 24.1 days), a remarkable total body weight loss (TBWL) of 45.3 kg was observed, with mean body weight decreasing from 129.8 to 83.6 kg (*p*-value < 0.001, Cohen’s d = 2.18). Thus, mean BMI significantly decreased from 47.7 kg/m^2^ (severe obesity) to 30.9 kg/m^2^ (overweight category) (*p*-value < 0.001, Cohen’s d = 2.35). This translated into a mean %EWL of 63.9%. At the beginning of the study, 15.5% of the patients had obesity class II and 84.5% had obesity class III. After surgery, 20.7% of the cohort achieved normal weight (Table 1). Student’s *t*-tests demonstrated a statistically significant reduction in both circulating total lipids (TLs) (from 6.6 to 5.1 mg/mL; *p* < 0.001, Cohen’s d = 1.12) and fasting glycemia (from 119.0 to 92.3 mg/dL; *p* < 0.001, Cohen’s d = 0.93). A significant decrease in the frequency of artery hypertension (AHT) was also observed (*p*-value = 0.007).

The cohort was segmented by age, sex, diabetes mellitus condition, AHT condition, and family history of obesity (obesity in first-degree relatives). Differences between groups before surgery are shown in Table S2. Briefly, total lipids were significantly higher in men (*p*-value = 0.02, rrb = 0.33) and diabetic patients (*p*-value = 0.003, rrb = 0.43), indicating moderate effect sizes.

Differences between groups after surgery are shown in Table S3. Younger patients exhibited significantly lower blood glucose (*p* = 0.001, rrb = 0.62) and TL levels (*p* = 0.013, rrb = 0.34), indicating strong and moderate effects, respectively. BMI was also significantly reduced in this group (*p* = 0.001, Cohen’s d = −0.86), with a notably higher %EWL (*p* = 0.004), suggesting greater weight loss outcomes. BMI was significantly higher among patients with a family history of obesity (*p*-value = 0.03, Cohen’s d = −0.59) and those with AHT (*p*-value = 0.008). Patients showing AHT had higher levels of blood glucose (*p*-value = 0.004, rrb = 0.65) and lower %EWL (*p*-value = 0.008, rrb = 0.44), displaying greater weight loss. No sex differences were found after surgery.

### 3.2. Effect of Bariatric Surgery on Serum Levels of Inorganic Contaminants

A total of 39 out of 55 analyzed elements were detected, either before or after surgery, in at least one patient (Table 2). Detection rates significantly decreased after surgery in the case of barium, chromium, gallium, palladium, platinum, and silicon (*p*-value < 0.05). Serum concentrations of gold (*p*-value = 0.030, Cohen’s d = −0.123), copper (*p*-value < 0.001, rrb = 0.91), mercury (*p*-value < 0.001, Cohen’s d = 0.85), platinum (*p*-value = 0.034, Cohen’s d = −3.06), and selenium (*p*-value < 0.001, Cohen’s d = 1.12) significantly decreased after surgery. Conversely, serum concentrations of iron and zinc significantly increased after surgery (*p*-value = 0.044 and 0.001, respectively).

Differences between groups, in relation to the concentration of organic elements before and after surgery, were also analyzed (Tables S2 and S3). Before surgery, iron serum levels were higher in men (*p*-value = 0.016) while copper serum levels were higher in women (*p*-value = 0.002, rrb = 0.53). In patients younger than 43 (n = 30, 51.7%), analyses revealed lower strontium concentrations compared with older patients (*p*-value = 0.015). Patients with diabetes showed higher serum levels of platinum and zinc (*p*-values = 0.007 and 0.012, respectively; Table S2). Finally, patients with a family history of obesity showed higher serum levels of selenium (*p*-value < 0.001).

After surgery, women exhibited significantly higher copper serum levels (*p*-value < 0.001, rrb = −0.62). Regarding age, patients older than 43 years old showed higher serum levels of bromine, selenium, and zinc (*p*-value = 0.012, 0.023, and < 0.001, respectively; Table S3). Serum levels of bromine and rubidium were higher among patients with diabetes (*p*-value = 0.002 and 0.021, respectively), while serum levels of strontium were lower (*p*-value = 0.009, rrb = 0.41). Serum levels of rubidium were also higher among patients with AHT (*p*-value = 0.010, Cohen’s d = −0.79). Finally, maternal obesity was linked to significantly higher cobalt and zinc serum levels (*p*-value = 0.018, rrb = 0.59, and *p*-value = 0.004, Cohen’s d = −0.89, respectively), reflecting strong to very strong effects.

**Figure 1:**
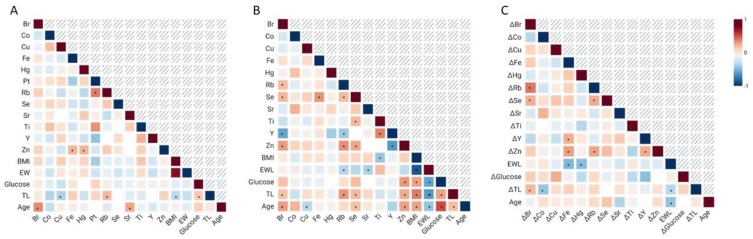
Correlations between serum levels of inorganic elements and sociodemographic variables before bariatric surgery (**A**), after bariatric surgery (**B**), and those observed in relation to the variation in serum concentrations of inorganic elements, defined as the concentration after surgery minus the concentration before surgery (**C**). * highlights significant correlations.

Correlation tests were performed to identify possible associations between the inorganic elements whose detection rates were above 30%, as well as correlations with demographic and clinical variables. Before surgery, age positively correlated with bromine and strontium (Pearson’s r = 0.483 and Spearman’s rho = 0.396; *p*-value = 0.001 and 0.006, respectively) (Figure 1A; Table S4). Serum copper negatively correlated with TL (Spearman’s rho = −0.316; *p*-value = 0.030), while rubidium showed a positive correlation with TL (Spearman’s rho = 0.325; *p*-value = 0.026). Zinc concentration positively correlated with iron and mercury (Spearman’s rho = 0.341 and 0.348; *p*-value = 0.020 and 0.026, respectively), and rubidium positively correlated with platinum (Spearman’s rho = 0.525; *p*-value = 0.047).

Post-surgery, bromine levels correlated with TL (Spearman’s rho = 0.289; *p*-value = 0.042) and age (Pearson’s r = 0.424; *p*-value = 0.002). Tests also revealed significant correlations between copper and age (Spearman’s rho = −0.285; *p*-value = 0.045). Rubidium correlated with %EWL (Pearson’s r = −0.287; *p*-value = 0.043) and TL (Spearman’s rho = 0.442; *p*-value = 0.001). Selenium correlated with TL (Spearman’s rho = 0.327; *p*-value = 0.020) and age (Pearson’s r = 0.303; *p*-value = 0.032) and titanium concentration correlated with BMI (Spearman’s rho = −0.378; *p*-value = 0.024). Correlations between zinc levels and blood glucose (Spearman’s rho = 0.444; *p*-value = 0.020), TL (Spearman’s rho = 0.375; *p*-value = 0.007), and age (Spearman’s rho = 0.413; *p*-value = 0.003) were observed (Figure 1B; Table S5). Levels of bromine showed positive correlations with rubidium (Pearson’s r = 0.314; *p*-value = 0.026), selenium (Pearson’s r = 0.337; *p*-value = 0.017), and zinc (Spearman’s rho = 0.429; *p*-value = 0.002), as well as a negative correlation with yttrium (Spearman’s rho = −0.534; *p*-value = 0.001). Tests also showed correlations between rubidium and selenium (Pearson’s r = 0.320; *p*-value = 0.023), yttrium (Spearman’s rho = −0.369; *p*-value = 0.032), and zinc (Spearman’s rho = 0.462; *p*-value = 0.001). In parallel, selenium positively correlated with iron (Spearman’s rho = 0.452; *p*-value = 0.001), titanium (Spearman’s rho = 0.334; *p*-value = 0.047), and zinc (Spearman’s rho = 0.390; *p*-value = 0.005). Finally, correlations between titanium and yttrium (Spearman’s rho = 0.503; *p*-value = 0.011) and between yttrium and zinc (Spearman’s rho = −0.577; *p*-value < 0.001) were found (Figure 1B; Table S5). Of note, correlations for platinum were not further analyzed due to its low detection rate after surgery.

Correlation analyses of the changes in concentration and sociodemographic variables displayed positive associations between Δ-bromine and Δ-TL (Spearman’s rho = 0.326; *p*-value = 0.043). Δ-cobalt correlated with Δ-TL (Spearman’s rho = −0.378; *p*-value = 0.040). Δ-iron correlated with %EWL (Spearman’s rho = −0.448; *p*-value = 0.005). Lastly, a negative correlation between Δ-mercury and %EWL (Spearman’s rho = −0.422; *p*-value = 0.009) was also found (Figure 1C; Table S6). Analyses of the changes in the serum concentration of inorganic elements revealed that Δ-bromine was positively correlated with Δ-rubidium (Pearson’s r = 0.537; *p*-value < 0.001) and Δ-selenium (Pearson’s r = 0.365; *p*-value = 0.022). Associations between Δ-iron and Δ-yttrium (Spearman’s rho = 0.369; *p*-value = 0.030) and between Δ-iron and Δ-zinc (Spearman’s rho = 0.417; *p*-value = 0.009) were also identified. Δ-rubidium positively correlated with Δ-selenium (Pearson’s r = 0.369; *p*-value = 0.021) and Δ-zinc (Pearson’s r = 0.400; *p*-value = 0.012) and Δ-yttrium was positively correlated with Δ-zinc (Pearson’s r = 0.372; *p*-value = 0.028) (Figure 1C; Table S6).

## 4. Discussion

Of the 55 elements analyzed, 70.9% (39 elements) were detected in at least one individual before or after bariatric surgery. Of these, serum concentrations of seven elements (17.9%) underwent significant alterations following bariatric surgery, with gold, copper, mercury, platinum, and selenium exhibiting decreases, while iron and zinc levels increased.

Obesity leads to significant disruptions in the homeostasis of trace and other elements. Research has shown that obese individuals exhibit decreased levels of zinc, iron, selenium, and strontium, while levels of copper, chromium, and manganese are significantly increased [32,33]. This disruption seems to contribute to metabolic and oxidative stress [32,33]. While weight loss can ameliorate some of these disruptions, it may also lead to the release of stored compounds, potentially impacting metabolic function. Moreover, bariatric surgery has the potential to influence the serum concentrations of a wide range of inorganic elements, including heavy metals. Such surgical interventions may disrupt the homeostatic balance of essential metals by altering their absorption and metabolism. Specifically, while copper and zinc concentrations remain relatively stable, selenium shows a tendency to decline post-surgery [34,35]. However, while the observed changes in serum element concentrations are notable, further exploration reveals a more intricate interplay at the molecular level, including alterations in isotopic composition. Thus, bariatric surgery can alter the isotopic compositions of essential metals like copper, zinc, and iron, reflecting changes in their physiological processing rather than their concentrations [34,35]. In the present study, serum levels of copper, iron, and selenium were altered following surgery and rapid weight loss, reinforcing previous findings and suggesting a complex relationship with adipose tissue. The impact of bariatric surgery transcends weight loss and its immediate consequences on biochemical and clinical parameters such as total lipid levels and hypertension risk. By altering intestinal absorption mechanisms, this surgical procedure can significantly modify the body’s elemental profile [36]. In routine clinical practice, patients who have undergone such surgery are recommended to take dietary supplements to maintain the homeostasis of trace elements and essential elements [35]. In this regard, iron and zinc, two elements commonly found in mineral supplements, were the only substances whose levels increased significantly after surgery. This increase may be attributed to physician-prescribed supplementation. However, this hypothesis cannot be confirmed as we do not have information regarding these prescriptions. On the contrary, the observed decreases in certain elements post-surgery can be attributed to the reduced absorption of these elements, particularly given that many are dietary in origin and, unlike lipophilic compounds [26], are not extensively stored in adipose tissue unless certain biotransformations increase the lipophilic characteristics of specific compounds (i.e., methylmercury). Due to its toxicological significance, the observed decrease in mercury concentration following surgery warrants special attention. A study on Norwegian obese patients undergoing bariatric surgery found that, 12 months post-surgery, the median whole-blood mercury concentration decreased by 31% [37]. In the present study, after a mean follow-up of 534 days, the median concentration decreased by 50%, highlighting the fact that the detection frequency did not change (87.2 and 84.0% before and after surgery, respectively). The underlying causes of this reduction remain unclear. Considering the relatively short half-life of mercury (≈50 days) [38], the observed reduction levels may be attributed to decreased fish and seafood consumption following surgery, as these are major contributors to mercury body burden [39].

We observed that age and sex were demographic factors associated with the concentration of certain elements. Specifically, bromide, selenium, and zinc were higher in older patients who underwent bariatric surgery. In general, serum levels of selenium and zinc show that these micronutrient levels tend to decrease with advancing age [40]. However, in a Spanish adult population, age was positively associated with serum selenium levels, suggesting that selenium levels might initially increase and then decline in very old age [41]. For analyses related to the influence of age on serum levels of the elements, the median of the age distribution, which was 43 years, was used as a cut-off point. This age is far from being considered an “advanced age”. Thus, our results support previous observations. Interestingly, in a cohort of middle-aged and older Chinese adults, higher selenium levels were unexpectedly associated with increased risk factors for metabolic syndrome, suggesting that selenium status may not only decline with age but also play a more complex role in metabolic health [42]. This finding, along with the observed increased selenium concentration after bariatric surgery in the older population, focuses attention on this compound in relation to treatment failures, specifically weight regain post-surgery.

In relation to the influence that sex may exert on changes in element concentrations, our results have shown that, before surgery, iron levels were higher in men, and copper levels were higher in women. These findings are consistent with the existing literature, which reports higher copper levels in women [43]. Although women generally have higher serum iron levels, they also have a higher prevalence of anemia due to menstrual cycles, ultimately resulting in lower iron levels [44]. After surgery, only copper levels were significantly higher in women, supporting the hypothesis of mineral supplementation in bariatric surgery patients.

Associations and correlations were observed between some of the elements analyzed and medical parameters such as hypertension, diabetes, blood glucose, and total lipids. Prior to surgery, individuals with diabetes exhibited elevated serum zinc levels. After surgery, a significant positive correlation emerged between serum zinc concentrations and glucose levels. In line with the present results, previous studies have demonstrated a linear association between elevated serum zinc levels and an increased risk of both prediabetes and diabetes [45]. Moreover, lower serum zinc levels have been associated with poorer glycemic control in type 2 diabetes patients. Our findings suggest a potential link between zinc status and glucose homeostasis, highlighting the importance of zinc in glucose metabolism.

A post-surgical increase in serum rubidium levels was observed in hypertensive patients and patients with diabetes. The role of rubidium in diabetes remains unclear, as there are studies supporting this association [46] and others that have found no association [47]. Regarding hypertension, no previous association with serum rubidium levels has been described. However, associations between serum levels of other inorganic elements, such as lead, and the risk of hypertension and stroke have been previously reported [48].

Finally, a significant positive correlation was observed between total lipid levels and the serum concentrations of bromine, rubidium, selenium, and zinc, particularly post-operatively. Given that bromine is a constituent of widely used compounds such as polybrominated flame retardants, the metabolic fate of these compounds may influence serum bromine concentrations. While a positive correlation between total lipids and bromine levels was reported, the specific role of polybrominated biphenyls in this relationship remains complex [49,50]. Although the relationship between serum rubidium levels and total lipids remains unexplored, the literature consistently reports a positive correlation between selenium levels and lipid profiles [51,52,53]. Our findings corroborate these previous observations, further emphasizing the role of selenium in lipid metabolism and obesity. Regarding zinc, a positive correlation exists with the serum lipid profile [54,55]. Interestingly, zinc supplementation has been shown to significantly reduce serum cholesterol and triglyceride levels [56,57], positioning zinc as a central element in the context of obesity and highlighting the role of micronutrient supplementation in obesity and pre- or post-bariatric surgery interventions.

This study has several limitations that must be considered when interpreting the results. Firstly, there are limitations inherent to experimental design. The longitudinal design of this study is subjected to limitations related to sample selection and time-related factors. Secondly, there are limitations related to the influence of factors that determine the presence of these elements in the body. In this regard, the influence of the pre-intervention diet, as well as the post-surgical dietary intervention, will condition the intake of inorganic elements such as mercury. Additionally, certain toxic habits, particularly smoking, and the intensity and duration thereof, may exert a significant influence. Given the lack of detailed information regarding these variables, our results cannot be interpreted within these contexts. Certain medical prescriptions, specifically those related to dietary supplements post-surgery, may influence the serum levels of some of the elements considered, especially trace elements and micronutrients. Given the absence of this information, the observed variations in relation to bariatric surgery must be considered in light of this limitation. Finally, given that some of the analyzed elements are lipophilic and considered endocrine disruptors, biomonitoring these elements in adipose tissue is necessary. Having the inorganic element profile of adipose tissue could help to elucidate some of the alterations observed in serum levels after bariatric surgery. On the other hand, this study has several strengths. Longitudinal studies offer a unique perspective on dynamic and changing processes, allowing for the identification of patterns, trends, and causal relationships. Additionally, it provides relevant information about the behavior and influence of more than fifty inorganic elements in the context of obesity and rapid weight loss, a poorly explored area of research. Although the sample size is relatively small, rigorous statistical analyses were conducted, considering, for example, elements detected in more than 30% of individuals for correlation analyses. This strengthens the reported results despite the limitations.

## 5. Conclusions

This study demonstrates that bariatric surgery induces notable alterations in serum inorganic element levels, highlighting the intricate relationship between these elements and lipid metabolism. The results underscore the significance of zinc and selenium in this context.

## Figures and Tables

**Table 1:** Physical condition and habits of the patients before and after bariatric surgery (mean follow-up = 534 days (SD = 24.1 days)).

Variables	Before Intervention (n = 58)	After Intervention (n = 56)	*p*-Value
Weight (kg)			
Mean ± SD	129.8 ± 24.4	83.6 ± 19.2	<0.001 ^†^
Median (range)	127.8 (93.2–189.1)	83.7 (54.0–134.3)	<0.001 ^††^
Body mass index (kg/m^2^)			
Mean ± SD	47.7 ± 6.5	30.9 ± 6.0	<0.001 ^†^
Median (range)	47.9 (35.5–59.4)	30.7 (20.5–44.0)	<0.001 ^††^
Normal weight (n, (%))	0 (0)	12 (20.7)	<0.001 *
Overweight (n, (%))	0 (0)	17 (29.3)	<0.001 *
Obesity class I (n, (%))	0 (0)	13 (22.4)	<0.001 *
Obesity class II (n, (%))	9 (15.5)	11 (19.0)	0.617 *
Obesity class III (n, (%))	49 (84.5)	5 (8.6)	<0.001 *
Total body weight loss (kg)			
Mean ± SD		45.3 ± 20.8	NA
Median (range)		43.4 (−0.6–92.1)	NA
% Excess weight loss			
Mean ± SD		63.9 ± 24.3	NA
Median (range)		65.5 (−1.4–102.8)	NA
Smoking habit (n, (%))			
Yes	10 (18.2)	14 (25.5)	0.133 *
No	33 (60.0)	36 (65.5)	
Former smoker	12 (21.8)	5 (9.1)	
Nº of cigarettes			
Mean ± SD	11.6 ± 6.3	15 ± 7.6	0.830 ^†^
Median (range)	11 (4–20)	12 (4–30)	0.855 ^††^
Total lipids (mg/mL)			
Mean ± SD	6.6 ± 0.9	5.1 ± 1.1	<0.001 ^†^
Median (range)	6.5 (4.9–10.0)	4.8 (2.0–8.2)	<0.001 ^††^
Artery hypertension (n, (%))			
Yes	25 (43.1)	13 (24.5)	0.007 *
No	33 (56.9)	40 (75.5)	
Glucose (mg/dL)			
Mean ± SD	119.0 ± 46.4	92.3 ± 17.6	<0.001 ^†^
Median (range)	103.0 (85.0–321.0)	92 (63.0–155.0)	<0.001 ^††^

Abbreviations: NA, not applicable; SD, standard deviation. ^†^ Student’s *t*-test. ^††^ Wilcoxon’s W test. * McNemar’s test.

**Table 2:** Detection rates and serum concentrations of inorganic elements before and after surgery. Concentrations are expressed as median (p^25th^–p^75th^).

Element	Before Surgery (n = 47)		After Surgery (n = 50)		ΔConcentration (ng/mL)	*p*-Value ^#^
	Detection Rate [N, (%)]	Concentration (ng/mL)	Detection Rate [N, (%)] ^+++^	Concentration (ng/mL)		
Al (Aluminum)	4 (8.5)	106.8 (33.0–735.9)	7 (14.0)	0.32 (0.32–39.1)	0.21 (−24.3–7.86)	ND
As (Arsenic)	0 (0.0)	ND	1 (2.0)	22.1 (22.1–22.1)	ND	ND
Au (Gold)	14 (29.8)	2.71 (1.69–3.43)	11 (22.0)	2.67 (1.82–3.61)	0.87 (−2.99–1.80)	0.030
Ba (Barium)	7 (14.9)	0.64 (0.45–2.22)	0 (0.0) *	ND	−0.55 (−1.93–(−0.40))	ND
Be (Beryllium)	13 (27.7)	3.39 (3.31–3.45)	7 (14.0)	2.35 (1.25–2.40)	−3.27 (−3.41–0.12)	ND
Br (Bromine)	47 (100.0)	18.4 (16.2–20.6) ^θ^	50 (100.0)	18.7 (15.4–21.8) ^θ^	−0.90 (−2.76–1.81) ^θ^	0.318
Cd (Cadmium)	2 (4.3)	0.20 (0.16–0.24)	8 (16.0)	0.29 (0.17–0.62)	0.21 (0.04–0.59)	ND
Ce (Cerium)	1 (2.1)	0.17 (0.17–0.17)	4 (8.0)	0.16 (0.07–0.27)	0.05 (0.04–0.23)	ND
Co (Cobalt)	28 (59.6)	0.27 (0.18–0.68)	25 (50.0)	0.18 (0.12–0.59)	−0.15 (−0.34–0.27)	0.842
Cr (Chromium)	9 (19.1)	2.18 (0.32–3.44)	1 (2.0) *	11.1 (11.1–11.1)	−2.15 (−3.42–(−0.30))	ND
Cs (Caesium)	1 (2.1)	0.34 (0.34–0.34)	7 (14.0)	0.24 (0.17–0.50)	0.26 (0.09–0.57)	ND
Cu (Copper)	47 (100.0)	1.30 (1.10–1.38) ^θ^	50 (100.0)	1.03 (0.82–1.15) ^θ^	−0.29 (−0.48–(−0.17)) ^θ^	<0.001 ^†^
Dy (Dysprosium)	1 (2.1)	0.06 (0.06–0.06)	1 (2.0)	0.06 (0.06–0.06)	0.00 (−0.02–0.02)	ND
Fe (Iron)	47 (100.0)	1.07 (0.88–1.42) ^θ^	50 (100.0)	1.34 (0.94–1.75) ^θ^	0.21 (−0.25–0.60) ^θ^	0.044 ^†^
Ga (Gallium)	8 (17)	0.30 (0.20–0.54)	0 (0.0) *	ND	−0.22 (−0.55–(−0.17))	ND
Gd (Gadolinium)	0 (0.0)	ND	3 (6.0)	0.07 (0.06–0.12)	0.05 (0.05–0.05)	ND
Hg (Mercury)	41 (87.2)	0.84 (0.65–1.52)	42 (84.0)	0.42 (0.16–0.75)	−0.57 (−0.68–(−0.01))	<0.001
In (Indium)	2 (4.3)	0.23 (0.18–0.29)	0 (0.0)	ND	−0.21 (−0.26–(−0.15))	ND
La (Lanthanum)	1 (2.1)	0.08 (0.08–0.08)	8 (16.0)	0.07 (0.05–0.14)	0.04 (0.03–0.09)	ND
Li (Lithium)	1 (2.1)	11.9 (11.9–11.9)	0 (0.0)	ND	ND	ND
Mn (Manganese)	4 (8.5)	1.70 (1.25–2.61)	14 (28.0)	1.87 (1.55–2.28)	1.57 (0.15–1.87)	0.760
Mo (Molybdenum)	0 (0.0)	ND	1 (2.0)	20.7 (20.7–20.7)	20.7 (20.7–20.7)	ND
Nd (Neodymium)	9 (19.1)	0.08 (0.06–0.10)	12 (24.0)	0.15 (0.13–0.16)	0.05 (−0.04–0.13)	0.287
Os (Osmium)	8 (17.0)	0.79 (0.74–0.90)	3 (6.0)	0.96 (0.85–1.27)	−0.72 (−0.76–0.16)	0.121
Pd (Palladium)	12 (25.5)	0.08 (0.07–0.10)	4 (8.0) *	0.05 (0.05–0.07)	−0.04 (−0.05–(−0.04))	0.577
Pr (Praseodymium)	0 (0.0)	ND	1 (2.0)	0.07 (0.07–0.07)	ND	ND
Pt (Platinum)	15 (31.9)	0.13 (0.08–0.16)	3 (6.0) *	0.13 (0.13–0.16)	−0.11 (−0.14–0.00)	0.034
Rb (Rubidium)	47 (100.0)	151.4 (132.2–168.3)	50 (100.0)	148.4 (129.6–167.7)	0.92 (−20.5–17.2)	0.720
Ru (Ruthenium)	0 (0.0)	ND	1 (2.0)	0.06 (0.06–0.06)	0.04 (0.04–0.04)	ND
Se (Selenium)	47 (100.0)	87.8 (75.4–93.5)	50 (100.0)	57.3 (45.8–73.9)	−25 (−42.6–(−11.5))	<0.001
Si (Silicon)	5 (10.6)	496.7 (329.4–785.4)	1 (2.0) *	353.4 (353.4–353.4)	−432 (−496.7–(−329.4))	ND
Sr (Strontium)	47 (100.0)	23.5 (17.8–29.8)	50 (100.0)	23.8 (19.6–27.9)	1.66 (−3.72–4.23)	0.694
Ta (Tantalum)	9 (19.1)	0.09 (0.09–0.13)	20 (40.0)	0.07 (0.06–0.10)	0.03 (−0.05–0.05)	0.601
Th (Thorium)	5 (10.6)	0.08 (0.08–0.22)	16 (32.0)	0.09 (0.06–0.14)	0.03 (−0.04–0.07)	ND
Ti (Titanium)	30 (63.8)	7.30 (5.84–9.69)	36 (72.0)	8.34 (6.50–11.2)	1.28 (−5.19–6.83)	0.060
U (Uranium)	3 (6.4)	0.25 (0.16–0.48)	5 (10.0)	0.25 (0.23–0.81)	0.08 (−0.14–0.5)	ND
Y (Yttrium)	22 (46.8)	0.12 (0.08–0.14)	34 (68.0)	0.10 (0.08–0.15)	0.05 (−0.05–0.08)	0.503
Yb (Ytterbium)	0 (0.0)	ND	1 (2.0)	0.06 (0.06–0.06)	ND	ND
Zn (Zinc)	47 (100.0)	469.6 (310.3–570.5)	50 (100.0)	556.8 (468.3–639.3)	99.4 (−39.0–277.9)	0.001

^#^ Student’s *t*-test. ^†^ Wilcoxon’s W test. Cu and Fe did not adjust to normal distribution (Shapiro–Wilk). ^+++^ McNemar’s test. * *p* < 0.05. ^θ^ Concentration expressed in µg/mL. ND: differences were not determined through paired-sample *t*-tests due to the lack of coincident measures before and after surgery.

## Data Availability

All data will be available upon request.

## Supplementary Materials

The following supporting information can be downloaded at https://www.mdpi.com/article/10.3390/toxics13030152/s1: Table S1: Limit of detection (LOD) and limit of quantification (LOQ) of the analyzed elements (n = 55) expressed in ppb; Table S2: Significant differences between groups before surgery; Table S3: Significant differences between groups after surgery; Table S4: Correlations between serum inorganic elements and sociodemographic variables before surgery; Table S5: Correlations between serum inorganic elements and sociodemographic variables after surgery; Table S6: Correlations between the variation in serum inorganic elements and sociodemographic variables.
