# Supplementary material for: Biomonitoring of Serum Inorganic Element Concentrations in Morbidly Obese Patients: Impact of Bariatric Surgery
Source: Toxics. 2025 Feb 23;13(3):152. doi: 10.3390/toxics13030152 (PMC11945562; doi:10.3390/toxics13030152)
Supplement: Supplementary file 1 [file toxics-13-00152-s001.zip › Table S2.pdf]

Table S2. Significant differences between groups before surgery.

	Variable	Groups	Mean ± SD	Median ($p25^{th}$ - $p75^{th}$)	p-value
Age* (Years)	Sr (ng/mL)	<43	21.62 ± 7.067	22.29 (15.30 - 24.56)	0.015[a]
		>43	26.44 ± 7.70	23.92 (21.22 - 33.81)	
Sex[1]	Cu (µg/mL)	Male	1.106 ± 0.225$^{\theta}$	1.069 (0.967 - 1.303)	0.002[b]
		Female	1.443 ± 0.480$^{\theta}$	1.319 (1.186 - 1.422)	
	Fe (µg/mL)	Male	1.271 ± 0.245$^{\theta}$	1.398 (1.050 - 1.480)	0.016[b]
		Female	1.155 ± 0.585$^{\theta}$	1.014 (0.801 - 1.323)	
	TL (mg/mL)	Male	7.009 ± 0.993	6.473 (6.473 - 7.222)	0.025[b]
		Female	6.424 ± 0.846	6.473 (5.982 - 6.612)	
Diabetes[2]	Pt (ng/mL)	Non diabetic	0.113 ± 0.050	0.092 (0.074 - 0.160)	0.007[a]
		Diabetic	0.259 ± 0.172	0.259 (0.198 - 0.319)	
	TL (mg/mL)	Non diabetic	6.386 ± 0.888	6.473 (5.900 - 6.473)	0.003[b]
		Diabetic	6.941 ± 0.903	6.745 (6.473 - 7.196)	
	Zn (ng/mL)	Non diabetic	401.6 ± 158.9	370.6 (296.0 - 526.8)	0.012[a]
		Diabetic	519.2 ± 180.4	570.5 (428.6 - 611.4)	
AHT[3]	Glucose (mg/dL)	No	107.3 ± 29.77	101.0 (93.00 - 115.0)	0.019[b]
		Yes	134.4 ± 59.05	108.0 (100.0 - 154.0)	
Family history[4]	Se (ng/mL)	No	75.82 ± 17.56	75.38 (65.26 - 92.10)	<0.001[a]
		Yes	90.95 ± 9.761	88.95 (86.80 - 96.83)	

Abbreviations: AHT, Artery Hypertension¸ TL, Total Lipids.

[a]Students t test.
[b]Mann-Whitney U test.
*Age was segmented according to the median distribution (<43 years (n = 30, 51.7%); ≥43 years (n = 28, 48.3%)).
[1]Female (n = 42, 72.4%); Male (n = 16, 27.6%).
[2]Non-diabetic (n = 36, 63.2%); diabetic (n = 21, 36.8%); 1 missing value.
[3]No AHT (n = 33, 56.9%); AHT (n = 25, 43.1%).
[4]No (n = 27, 51.9%); Yes (n = 25, 48.1%); 6 missing values.
