# Supplementary material for: Biomonitoring of Serum Inorganic Element Concentrations in Morbidly Obese Patients: Impact of Bariatric Surgery
Source: Toxics. 2025 Feb 23;13(3):152. doi: 10.3390/toxics13030152 (PMC11945562; doi:10.3390/toxics13030152)
Supplement: Supplementary file 1 [file toxics-13-00152-s001.zip › Table S3.pdf]

Table S3. Significant differences between groups after surgery.

	Variable	Groups	Mean ± SD	Median (p25th - p75th)	p-value
Age*	Br (µg/mL)	<43	17.17 ± 4.044$^{\theta}$	17.23 (14.19 - 19.86)	0.012[a]
		>43	19.66 ± 3.475$^{\theta}$	19.05 (16.75 - 22.15)	
	Glucose	<43	83.73 ± 8.548	85.00 (77.00 - 87.50)	0.001[b]
		>43	99.94 ± 20.15	97.00 (92.00 - 107.0)	
	BMI (kg/m^2)	<43	28.60 ± 5.639	27.86 (24.95 - 31.20)	0.001[a]
		>43	33.38 ± 5.507	33.45 (29.34 - 37.16)	
	EWL (%)	<43	72.13 ± 23.35	74.11 (60.63 - 88.51)	0.004[a]
		>43	55.12 ± 22.42	52.67 (42.22 - 69.61)	
	Se (ng/mL)	<43	52.26 ± 20.34	50.86 (41.46 - 66.52)	0.023[a]
		>43	63.97 ± 20.20	68.21 (54.32 - 78.28)	
	TL (mg/mL)	<43	4.718 ± 0.996	4.623 (4.226 - 5.000)	0.013[b]
		>43	5.410 ± 1.138	5.000 (4.716 - 5.822)	
	Zn (ng/mL)	<43	497.7 ± 103.2	407.6 (401.1 - 567.8)	<0.001[a]
		>43	619.0 ± 149.4	601.2 (515.6 - 653.0)	
Sex[1]	Cu (µg/mL)	Male	0.834 ± 0.148$^{\theta}$	0.776 (0.749 - 0.969)	<0.001[b]
		Female	1.124 ± 0.385$^{\theta}$	1.081 (0.931 - 1.227)	
Diabetes[2]	Br (µg/mL)	Non diabetic	17.60 ± 3.895$^{\theta}$	17.38 (14.68 - 20.23)	0.002[a]
		Diabetic	20.02 ± 3.740$^{\theta}$	20.02 (17.28 - 22.14)	
	Rb (ng/mL)	Non diabetic	142.2 ± 25.19	140.4 (127.5 - 160.2)	0.021[a]
		Diabetic	158.5 ± 27.59	158.7 (148.5 - 173.2)	
	Sr (ng/mL)	Non diabetic	26.72 ± 9.406	25.16 (21.21 - 29.19)	0.009[b]
		Diabetic	21.95 ± 8.269	19.67 (17.23 - 23.55)	
AHT[3]	Glucose (mg/dL)	No	88.10 ± 19.42	85.00 (76.50 - 94.75)	0.004[b]
		Yes	103.4 ± 11.87	101.5 (93.00 - 109.8)	
	BMI (kg/m^2)	No	29.67 ± 5.877	29.13 (25.05 - 34.70)	0.008[a]
		Yes	34.30 ± 5.754	32.95 (30.53 - 38.43)	
	EWL (%)	No	69.39 ± 23.34	70.73 (56.41 - 88.31)	0.008[b]
		Yes	50.93 ± 22.64	51.90 (37.50 - 68.71)	
	Rb (ng/mL)	No	143.6 ± 26.09	147.7 (128.0 - 161.0)	0.010[a]
		Yes	164.1 ± 25.23	164.1 (153.9 - 174.1)	
	TL (mg/mL)	No	4.962 ± 1.031	4.716 (4.313 - 5.048)	0.018[b]
		Yes	5.575 ± 1.111	5.000 (4.851 - 6.509)	
Family history[4]	BMI (kg/m^2)	No	29.24 ± 6.063	27.95 (25.09 - 32.79)	0.020[a]
		Yes	32.79 ± 5.909	32.39 (29.01 - 35.92)	
Maternal obesity[5]	Co (ng/mL)	No	0.235 ± 0.151	0.151 (0.078 - 0.300)	0.018[b]
		Yes	0.652 ± 0.569	0.622 (0.292 - 0.650)	
	Zn (ng/mL)	No	514.9 ± 110.1	509.3 (425.2 - 597.6)	0.004[a]
		Yes	634.1 ± 180.5	582.5 (539.4 - 683.9)	

Abbreviations: AHT, Artery Hypertension; BMI, Body Mass Index; EWL, Excess Weight Loss; TL, Total Lipids.

[a]Students t test.

[b]Mann-Whitney U test.

*Age was segmented according to the median distribution (<43 years (n = 30, 51.7%); ≥43 years (n = 28, 48.3%)).

[1]Female (n = 42, 72.4%); Male (n = 16, 27.6%).

[2]Non-diabetic (n = 36, 63.2%); diabetic (n = 21, 36.8%); 1 missing value.

[3]No AHT (n = 33, 56.9%); AHT (n = 25, 43.1%).

[4]No (n = 27, 51.9%); Yes (n = 25, 48.1%); 6 missing values.

[5]No (n = 36, 69.2%); Yes (n = 16, 30.8%); 6 missing values.
