# Supplementary material for: Biomonitoring of Serum Inorganic Element Concentrations in Morbidly Obese Patients: Impact of Bariatric Surgery
Source: Toxics. 2025 Feb 23;13(3):152. doi: 10.3390/toxics13030152 (PMC11945562; doi:10.3390/toxics13030152)
Supplement: Supplementary file 1 [file toxics-13-00152-s001.zip › Table S4.pdf]

Table S4. Correlations between serum inorganic elements and sociodemographic variables before surgery.

		Br	Co	Cu	Fe	Hg	Pt	Rb	Se	Sr	Ti	Y	Zn	BMI	EW	Glucose	TL	Age
Br	Est.	—																
	p-value	—																
Co	Est.	-0.181	—															
	p-value	0.355[b]	—															
Cu	Est.	-0.038	0.307	—														
	p-value	0.802[b]	0.113[b]	—														
Fe	Est.	0.119	-0.053	-0.148	—													
	p-value	0.423[b]	0.788[b]	0.319[b]	—													
Hg	Est.	-0.077	-0.122	0.020	0.064	—												
	p-value	0.630[b]	0.543[b]	0.900[b]	0.690[b]	—												
Pt	Est.	0.093	0.160	0.200	-0.346	0.204	—											
	p-value	0.743[b]	0.584[b]	0.474[b]	0.206[b]	0.466[b]	—											
Rb	Est.	0.243	-0.037	-0.220	0.022	-0.267	0.525	—										
	p-value	0.100[a]	0.853[b]	0.137[b]	0.882[b]	0.092[b]	**0.047[b]**	—										
Se	Est.	0.093	0.206	0.156	-0.189	-0.220	0.036	0.270	—									
	p-value	0.534[a]	0.292[b]	0.294[b]	0.203[b]	0.167[b]	0.903[b]	0.066[a]	—									
Sr	Est.	-0.055	0.112	-0.029	-0.079	0.256	0.021	-0.232	0.072	—								
	p-value	0.711[b]	0.570[b]	0.844[b]	0.595[b]	0.106[b]	0.944[b]	0.117[b]	0.630[b]	—								
Ti	Est.	0.035	0.289	0.142	-0.177	0.051	0.455	-0.076	-0.027	0.244	—							
	p-value	0.853[a]	0.192[b]	0.452[b]	0.347[b]	0.799[b]	0.163[b]	0.690[a]	0.888[a]	0.192[b]	—							
Y	Est.	-0.086	-0.179	0.212	-0.068	-0.202	-0.400	-0.030	0.173	-0.014	0.355	—						
	p-value	0.703[a]	0.713[b]	0.343[b]	0.762[b]	0.406[b]	0.750[b]	0.894[a]	0.441[a]	0.952[b]	0.284[a]	—						
Zn	Est.	0.211	0.041	-0.187	0.341	0.348	-0.164	0.255	0.123	-0.023	0.240	-0.003	—					
	p-value	0.155[a]	0.838[b]	0.206[b]	**0.020[b]**	**0.026[b]**	0.558[b]	0.084[a]	0.409[a]	0.876[b]	0.201[a]	0.988[a]	—					

BMI	Est.	0.199	-0.135	0.243	0.073	0.222	-0.343	-0.095	-0.101	-0.086	0.269	-0.176	0.062	—				
	p-value	0.180[a]	0.491[b]	0.100[b]	0.625[b]	0.162[b]	0.211[b]	0.525[a]	0.497[a]	0.563[b]	0.150[a]	0.434[a]	0.680[a]	—				
EW	Est.	0.031	-0.200	0.128	0.059	0.163	-0.193	-0.084	-0.183	-0.119	0.182	-0.215	-0.078	0.897	—			
	p-value	0.833[b]	0.307[b]	0.390[b]	0.692[b]	0.308[b]	0.490[b]	0.575[b]	0.217[b]	0.425[b]	0.334[b]	0.335[b]	0.603[b]	**<0.001[b]**	—			
Glucose	Est.	0.098	-0.118	-0.207	0.137	-0.015	0.216	0.027	-0.112	-0.051	-0.004	-0.040	-0.005	-0.117	-0.109	—		
	p-value	0.513[b]	0.550[b]	0.163[b]	0.358[b]	0.927[b]	0.439[b]	0.855[b]	0.452[b]	0.732[b]	0.983[b]	0.861[b]	0.976[b]	0.381[b]	0.417[b]	—		
TL	Est.	0.104	-0.298	-0.316	0.242	-0.202	0.211	0.325	-0.013	-0.128	-0.119	-0.016	0.222	-0.266	-0.222	0.264	—	
	p-value	0.485[b]	0.124[b]	**0.030[b]**	0.102[b]	0.206[b]	0.450[b]	**0.026[b]**	0.929[b]	0.391[b]	0.530[b]	0.943[b]	0.134[b]	**0.043[b]**	0.094[b]	**0.045[b]**	—	
Age	Est.	0.483	-0.238	0.048	0.056	0.036	-0.020	-0.129	-0.013	0.396	-0.057	-0.153	-0.069	0.034	-0.108	0.239	0.113	—
	p-value	**0.001[a]**	0.222[b]	0.751[b]	0.709[b]	0.824[b]	0.945[b]	0.386[a]	0.928[a]	**0.006[b]**	0.763[a]	0.497[a]	0.644[a]	0.800[a]	0.421[b]	0.071[b]	0.400[b]	—

Abbreviations: BMI, Body Mass Index; EW, Excess Weight; TL, Total Lipids.

[a]Pearson's r correlation test. Significant correlations are highlighted in bold.

[b]Spearman's ρ correlation test. Significant correlations are highlighted in bold.
