# Supplementary material for: Biomonitoring of Serum Inorganic Element Concentrations in Morbidly Obese Patients: Impact of Bariatric Surgery
Source: Toxics. 2025 Feb 23;13(3):152. doi: 10.3390/toxics13030152 (PMC11945562; doi:10.3390/toxics13030152)
Supplement: Supplementary file 1 [file toxics-13-00152-s001.zip › Table S5.pdf]

Table S5. Correlations between serum inorganic elements and sociodemographic variables after surgery.

		Br	Co	Cu	Fe	Hg	Rb	Se	Sr	Ti	Y	Zn	BMI	EWL	Glucose	TL	Age
Br	Est.	—															
	p-value	—															
Co	Est.	-0.166	—														
	p-value	0.426[b]	—														
Cu	Est.	-0.121	-0.057	—													
	p-value	0.400[b]	0.787[b]	—													
Fe	Est.	0.151	-0.042	0.193	—												
	p-value	0.294[b]	0.841[b]	0.178[b]	—												
Hg	Est.	0.000	0.290	0.017	0.088	—											
	p-value	1.000[b]	0.191[b]	0.917[b]	0.579[b]	—											
Rb	Est.	0.314	-0.105	0.001	0.148	-0.182	—										
	p-value	**0.026[a]**	0.618[b]	0.996[b]	0.305[b]	0.249[b]	—										
Se	Est.	0.337	0.123	0.239	0.452	0.057	0.320	—									
	p-value	**0.017[a]**	0.556[b]	0.094[b]	**0.001[b]**	0.721[b]	**0.023[a]**	—									
Sr	Est.	-0.134	0.356	0.119	0.062	0.206	0.036	0.252	—								
	p-value	0.353[b]	0.081[b]	0.409[b]	0.670[b]	0.190[b]	0.806[b]	0.077[b]	—								
Ti	Est.	-0.216	0.047	-0.130	0.158	0.046	-0.079	0.334	0.038	—							
	p-value	0.206[b]	0.861[b]	0.448[b]	0.357[b]	0.809[b]	0.647[b]	**0.047[b]**	0.827[b]	—							
Y	Est.	-0.534	0.021	0.021	0.132	-0.353	-0.369	-0.031	0.059	0.503	—						
	p-value	**0.001[b]**	0.930[b]	0.905[b]	0.456[b]	0.066[b]	**0.032[b]**	0.863[b]	0.740[b]	**0.011[b]**	—						
Zn	Est.	0.429	0.215	0.209	0.163	-0.054	0.462	0.390	-0.024	-0.016	-0.577	—					
	p-value	**0.002[b]**	0.300[b]	0.144[b]	0.258[b]	0.735[b]	**0.001[b]**	**0.005[b]**	0.868[b]	0.925[b]	**<0.001[b]**	—					
BMI	Est.	0.101	-0.148	0.146	0.133	-0.042	0.201	0.193	0.202	-0.378	-0.054	0.229	—				
	p-value	0.484[a]	0.479[b]	0.312[b]	0.357[b]	0.793[b]	0.162[a]	0.179[a]	0.159[b]	**0.024[b]**	0.760[b]	0.110[b]	—				

EWL	Est.	0.010	0.064	-0.082	-0.103	0.030	-0.287	-0.277	-0.320	0.308	0.007	-0.253	-0.860	—			
	p-value	0.944[a]	0.761[b]	0.572[b]	0.475[b]	0.851[b]	**0.043[a]**	0.052[a]	**0.024[b]**	0.068[b]	0.968[b]	0.076[b]	**<0.001[a]**	—			
Glucose	Est.	0.298	-0.002	-0.095	-0.053	-0.010	0.166	0.313	-0.010	-0.070	-0.061	0.444	0.403	-0.529	—		
	p-value	0.131[b]	0.994[b]	0.637[b]	0.795[b]	0.964[b]	0.409[b]	0.112[b]	0.959[b]	0.782[b]	0.830[b]	**0.020[b]**	**0.027[b]**	**0.003[b]**	—		
TL	Est.	0.289	-0.209	0.165	0.127	-0.064	0.442	0.327	0.200	-0.180	-0.133	0.375	0.521	-0.573	0.405	—	
	p-value	**0.042[b]**	0.316[b]	0.253[b]	0.380[b]	0.688[b]	**0.001[b]**	**0.020[b]**	0.163[b]	0.294[b]	0.455[b]	**0.007[b]**	**<0.001[b]**	**<0.001[b]**	**0.022[b]**	—	
Age	Est.	0.424	0.340	-0.285	0.037	0.034	0.181	0.303	0.112	0.014	-0.305	0.413	0.403	-0.383	0.658	0.302	—
	p-value	**0.002[a]**	0.096[b]	**0.045[b]**	0.797[b]	0.832[b]	0.208[a]	**0.032[a]**	0.439[b]	0.936[b]	0.079[b]	**0.003[b]**	**0.002[a]**	**0.004[a]**	**<0.001[b]**	**0.021[b]**	—

Abbreviations: BMI, Body Mass Index; EWL, Excess Weight Loss; TL, Total Lipids.

[a]Pearson's r correlation test. Significant correlations are highlighted in bold.

[b]Spearman's ρ correlation test. Significant correlations are highlighted in bold.
