# Supplementary material for: Biomonitoring of Serum Inorganic Element Concentrations in Morbidly Obese Patients: Impact of Bariatric Surgery
Source: Toxics. 2025 Feb 23;13(3):152. doi: 10.3390/toxics13030152 (PMC11945562; doi:10.3390/toxics13030152)
Supplement: Supplementary file 1 [file toxics-13-00152-s001.zip › Table S6.pdf]

Table S6. Correlations between the variation of serum inorganic elements and sociodemographic variables.

		Δ-Br	Δ-Co	Δ-Cu	Δ-Fe	Δ-Hg	Δ-Rb	Δ-Se	Δ-Sr	Δ-Ti	Δ-Y	Δ-Zn	EWL	Δ-Glucose	Δ-TL	Age
Δ-Br	Est.	—														
	p-value	—														
Δ-Co	Est.	-0.282	—													
	p-value	0.131[a]	—													
Δ-Cu	Est.	0.209	0.142	—												
	p-value	0.202[b]	0.453[b]	—												
Δ-Fe	Est.	0.250	-0.089	-0.200	—											
	p-value	0.124[b]	0.638[b]	0.221[b]	—											
Δ-Hg	Est.	-0.147	-0.053	0.096	0.271	—										
	p-value	0.377[b]	0.784[b]	0.564[b]	0.100[b]	—										
Δ-Rb	Est.	0.537	-0.182	0.048	0.220	-0.191	—									
	p-value	**<0.001[a]**	0.337[a]	0.771[b]	0.178[b]	0.250[b]	—									
Δ-Se	Est.	0.365	0.160	0.283	0.250	-0.145	0.369	—								
	p-value	**0.022[a]**	0.399[a]	0.082[b]	0.125[b]	0.383[b]	**0.021[a]**	—								
Δ-Sr	Est.	-0.154	0.346	0.117	0.019	0.095	-0.161	0.138	—							
	p-value	0.349[a]	0.061[a]	0.475[b]	0.909[b]	0.571[b]	0.328[a]	0.401[a]	—							
Δ-Ti	Est.	-0.075	-0.018	-0.097	-0.043	-0.276	-0.076	0.066	-0.126	—						
	p-value	0.657[a]	0.928[a]	0.567[b]	0.798[b]	0.103[b]	0.655[a]	0.697[a]	0.457[a]	—						
Δ-Y	Est.	-0.267	0.082	-0.217	0.369	0.116	-0.119	0.182	0.265	0.048	—					
	p-value	0.121[a]	0.684[a]	0.210[b]	**0.030[b]**	0.513[b]	0.497[a]	0.295[a]	0.124[a]	0.786[a]	—					
Δ-Zn	Est.	0.242	0.026	-0.006	0.417	0.011	0.400	0.311	0.129	-0.300	0.372	—				
	p-value	0.137[a]	0.892[a]	0.969[b]	**0.009[b]**	0.947[b]	**0.012[a]**	0.054[a]	0.434[a]	0.072[a]	**0.028[a]**	—				
EWL	Est.	0.019	0.120	-0.106	-0.448	-0.422	-0.039	-0.067	-0.175	0.110	-0.132	-0.130	—			
	p-value	0.909[a]	0.527[a]	0.520[b]	**0.005[b]**	**0.009[b]**	0.811[a]	0.683[a]	0.286[a]	0.516[a]	0.448[a]	0.430[a]	—			

Δ-Glucose	Est.	0.249	0.180	0.367	0.031	-0.259	0.188	-0.059	0.100	-0.265	-0.416	-0.018	0.050	—		
	p-value	0.252[b]	0.461[b]	0.085[b]	0.890[b]	0.232[b]	0.390[b]	0.790[b]	0.649[b]	0.234[b]	0.061[b]	0.934[b]	0.794[b]	—		
Δ-TL	Est.	0.326	-0.378	0.169	0.230	0.070	0.198	-0.010	0.150	0.014	-0.071	0.142	-0.277	0.219	—	
	p-value	**0.043**[b]	**0.040**[b]	0.305[b]	0.159[b]	0.676[b]	0.226[b]	0.954[b]	0.362[b]	0.932[b]	0.686[b]	0.387[b]	**0.039**[b]	0.228[b]	—	
Age	Est.	-0.038	0.164	0.091	0.080	0.037	0.185	0.275	-0.301	-0.001	-0.031	0.171	-0.383	-0.026	0.112	—
	p-value	0.817[a]	0.386[a]	0.581[b]	0.630[b]	0.824[b]	0.259[a]	0.090[a]	0.062[a]	0.996[a]	0.861[a]	0.297[a]	**0.004**[a]	0.887[b]	0.404[b]	—

Abbreviations: BMI, Body Mass Index; EWL, Excess Weight Loss; TL, Total Lipids.

[a]Pearson's r correlation test. Significant correlations are highlighted in bold.

[b]Spearman's ρ correlation test. Significant correlations are highlighted in bold.
